# Endothelial-to-haematopoietic transition: an update on the process of making blood

**DOI:** 10.1042/BST20180320

**Published:** 2019-03-22

**Authors:** Katrin Ottersbach

**Affiliations:** MRC Centre for Regenerative Medicine, University of Edinburgh, Edinburgh EH16 4UU, U.K.

**Keywords:** aorta-gonads-mesonephros, developmental haematopoiesis, endothelial-to-haematopoietic transition, haematopoietic stem cells, haemogenic endothelium

## Abstract

The first definitive blood cells during embryogenesis are derived from endothelial cells in a highly conserved process known as endothelial-to-haematopoietic transition (EHT). This conversion involves activation of a haematopoietic transcriptional programme in a subset of endothelial cells in the major vasculature of the embryo, followed by major morphological changes that result in transitioning cells rounding up, breaking the tight junctions to neighbouring endothelial cells and adopting a haematopoietic fate. The whole process is co-ordinated by a complex interplay of key transcription factors and signalling pathways, with additional input from surrounding tissues. Diverse model systems, including mouse, chick and zebrafish embryos as well as differentiation of pluripotent cells *in vitro*, have contributed to the elucidation of the details of the EHT, which was greatly accelerated in recent years by sophisticated live imaging techniques and advances in transcriptional profiling, such as single-cell RNA-Seq. A detailed knowledge of these developmental events is required in order to be able to apply it to the generation of haematopoietic stem cells from pluripotent stem cells *in vitro* — an achievement which is of obvious clinical importance. The aim of this review is to summarise the latest findings and describe how these may have contributed towards achieving this goal.

## Introduction

**Figure 1.**
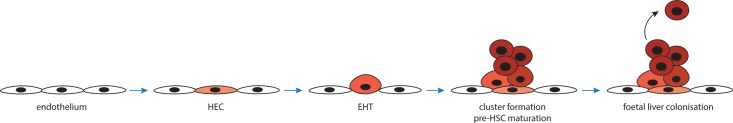
Stepwise formation of HSPCs from endothelial cells. Depicted are the morphological events thought to take place in the dorsal aorta of the mouse embryo during the formation of HSPCs. Up-regulation of a haematopoietic transcriptional programme in endothelial cells during the specification of HECs is marked by a light red colour. EHT is characterised by a shape change towards a rounder cell with further implementation of a haematopoietic programme and breaking of tight junctions with neighbouring endothelial cells. HECs giving rise to pro-HSCs and pre-HSCs is thought to result in the formation of intra-aortic clusters in which cells mature into haematopoietic stem and progenitor cells as they colonise the foetal liver.

In vertebrate animals, a close relationship exists between haematopoietic cells and endothelial cells. Haematopoietic cells travel through endothelium-lined blood vessels; they migrate in and out of the blood stream via docking to and transversing the endothelial layer; and haematopoietic stem and progenitor cells (HSPCs) are regulated and maintained by a vascular niche in the bone marrow during adult life and in the foetal liver during foetal life [1–3]. However, the relationship could not be any more intimate than during the generation of the first definitive HSPCs since these are directly produced from endothelial precursors in a process termed endothelial-to-haematopoietic transition (EHT). This cell fate transition involves switching on a haematopoietic transcriptional programme in select endothelial cells, which then become haemogenic, followed by considerable morphological changes that lead to the breaking of tight junctions with neighbouring endothelial cells and rounding up, to then being released into the blood stream (Figure 1).

The ontogeny of the haematopoietic system is characterised by multiple waves, multiple origins and shifting locations. The murine yolk sac gives rise to an initial wave of primitive erythroid cells, starting from embryonic day (E) 7.5, inside structures known as yolk sac blood islands, which consist of the first population of endothelial cells surrounding the newly formed primitive blood cells. The simultaneous appearance of blood and endothelial cells from a seemingly homogeneous cluster of mesodermal precursors initially led to the theory that primitive haematopoiesis did not originate from haemogenic endothelial cells (HECs), but from a developmentally earlier precursor termed haemangioblast, which displayed clonal bipotentiality for blood and endothelium and which had been detected *in vitro* [4] and *in vivo* [5]. More recent studies, however, have questioned the existence of such a bipotential precursor and have instead suggested that primitive blood may also derive from HECs located in the yolk sac [6,7].

In contrast with primitive haematopoiesis, definitive blood emerges in the context of a fully formed and functional vascular system. This again initiates in the yolk sac with the appearance of erythroid-myeloid progenitors (EMPs) by E8.5 [8] and immune-restricted progenitors [9]: two progenitor types that seem to complement each other with their differentiation potential. Adult-type haematopoietic stem cells (HSCs) that can fully repopulate the haematopoietic system of irradiated adult recipients upon direct transfer are not detected until E10.5. They first emerge in the dorsal aorta within the aorta-gonads-mesonephros (AGM) region and its associated major vessels, the umbilical and vitelline arteries [10–12]. Subsequently, they are also detected in the yolk sac, the placenta and the embryonic head, before they go on to colonise the foetal liver [13–15]. In each case, HSCs are found in the major vasculature within these tissues; however, it is not clear whether they are formed *de novo* in all of these tissues and whether a similar process of EHT is taking place. For example, even though embryonic head endothelial cells were shown to give rise to blood cells that contribute to the adult haematopoietic system [14], endothelium-associated haematopoietic cell clusters, which are signs of active EHT, have never been observed in the head [16].

Because of its importance as the first site of HSC generation, the process of EHT and its underlying molecular mechanisms have been almost exclusively studied in the dorsal aorta, which is what this review will therefore also concentrate on. Many excellent reviews on the haemogenic endothelium have been published in recent years [17–19], which this review will aim to provide an update on.

## First experimental evidence

The concept of an endothelial origin for definitive blood is not new. Histological observations made already 100 years ago proposed the existence of haemogenic endothelium [20–22]; however, experimental support came much later, initially in birds, but then also in mice. Using the avian system, which allows *ex vivo* manipulations, in this case, dye-labelling of endothelial cells prior to haematopoietic development, Jaffredo, Dieterlen-Lièvre and colleagues were able to demonstrate that the label was subsequently passed on to emerging blood cells [23]. Ten years later, temporally restricted genetic labelling allowed similar fate mapping in mice, which confirmed that endothelial cells gave rise to emerging blood cells in the dorsal aorta which then went on to colonise the foetal liver and the adult bone marrow, where they contributed to haematopoiesis long-term [24].

Advances in live imaging have allowed real-time observation of EHT [25–28]. Zebrafish embryos, which are transparent and develop externally, lend themselves particularly well to such observational studies. Stunning movies from the Herbomel lab of live zebrafish embryos revealed how endothelial cells with a flat morphology initiate EHT by rounding up, pulling neighbouring endothelial cells towards each other and eventually pinching off as round haematopoietic cells that are released into the circulation [28].

How these images can be reconciled with intra-aortic cluster formation is currently unknown. Marker expression analysis in mouse embryos suggests that emerging blood cells undergo further maturation within these clusters as they lose endothelial properties and up-regulate a haematopoietic programme. Indeed, an elegant explant culture system developed in the Medvinsky lab was able to dissect EHT into intermediate steps [29–31]. HECs were shown to mature into fully functional HSCs via a pro-HSC, pre-HSC I and pre-HSC II stage, which was accompanied by sequential up-regulation of the cell surface markers CD41, CD43 and CD45.

## Recreating EHT *in vitro*

A long-term goal, which could be considered the Holy Grail of the field due to its obvious clinical significance, is the generation of fully functional HSCs from pluripotent stem cells (PSCs) *in vitro*. While robust protocols exist for the differentiation of mouse and human PSCs [embryonic stem cells/induced PSCs (iPSCs)] into definitive haematopoietic cells, it is thought that these recapitulate the processes taking place in the early yolk sac and do not lead to the generation of HSCs. Indeed, differentiation of PSCs into considerable numbers of HSCs that are capable of long-term, high-level, multi-lineage haematopoietic reconstitution upon direct transplantation has proven to be a major challenge, although significant advances have been made recently [32,33].

What has become clear from the many attempts is the need for a thorough understanding of how HSCs are generated *in vivo* during embryogenesis, which has sparked renewed interest into the properties of HECs and the inner workings of the EHT (recently reviewed in refs. [34–36]). In fact, the PSC differentiation system with its accessibility and ease for manipulation has greatly contributed to our increased knowledge of the molecular details of the EHT.

Another approach for generating HSCs via recreating EHT *in vitro* that has shown great promise is the induction of a haemogenic programme in adult endothelial cells cultured within a vascular niche, which resulted in the production of HSCs with high-level, multi-lineage engraftment potential [37]. This study highlighted the fact that endothelial cells not only act as direct precursors of HSCs but are also components of the niche that supports the generation and maintenance of HSCs.

## Transcription factors—complexes, levels and dynamics

**Figure 2.**
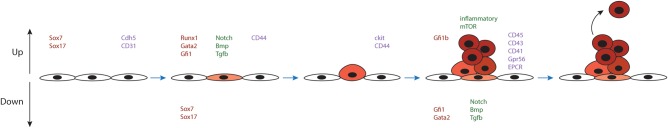
The regulation of EHT. Key transcription factors involved in the EHT are shown in red and key signalling pathways in green. Cell surface markers characteristic for each stage are shown in purple. Whether a gene is up-regulated or down-regulated during the process is highlighted by its position above or below the schematic, respectively.

Conversion of PSCs or adult ECs into HSCs has relied on the ectopic expression of key transcription factors that drive the endothelial-to-haematopoietic conversion by suppressing the endothelial transcriptional programme and instead switching on haematopoietic genes (Figure 2). Two key transcription factors that have been shown to be absolutely required for the generation of HSCs from HECs *in vivo* are Runx1 [28,38–40] and Gata2 [41–44]. Due to the technical difficulties associated with low cell numbers in developing embryos, little is currently known about the complexes and networks that these two transcription factors function in, although it was previously reported that two downstream targets of Runx1, the transcriptional repressors Gfi1 and Gfi1b, are also essential for successful completion of the EHT through repression of the endothelial programme via the recruitment of Kdm1a [45]. It is also known that the activity of Runx1 is absolutely dependent on the presence of the cofactor Cbfβ, with the knockout of Cbfb displaying an almost identical phenotype to Runx1 knockout embryos [46,47].

Considering the importance of Runx1 and Gata2 in the EHT, it may not come as a surprise that the timing and the levels of their expression need to be tightly controlled. Early indications came from studies showing that haploinsufficiency of Runx1 led to a temporal and spatial shift in HSC appearance [48], while half a dose of Gata2 resulted in reduced production of HSCs specifically in the AGM region [43]. Furthermore, it has been reported that HECs express lower levels of Runx1 than are expressed by haematopoietic progenitors [49].

Two Runx1 isoforms have been detected, with the Runx1b isoform being controlled by the proximal promoter P2, while the expression of the Runx1c isoform is under the control of the distal promoter P1. P2, and thus Runx1b, activity initiates earlier, during the initial stages of the EHT, while P1/Runx1c are activated later during the EHT and in already committed haematopoietic progenitors and remain more prominent in the adult haematopoietic system [50,51]. In addition, it was recently demonstrated, using the PSC system, that Runx1b levels need to be kept low for initiation and successful completion of the EHT, since increased levels of Runx1b accelerate the onset of EHT, but are unable to drive haematopoietic maturation [52].

Increased expression of Runx1 was also detected in embryos deficient for the Notch1 target genes *Hes1* and *Hes5* [53]. Despite these embryos containing larger intra-aortic clusters and increased numbers of phenotypic haematopoietic progenitors in the AGM, these were non-functional, and haematopoietic development was severely impaired. In this case, however, the authors linked the haematopoietic defect to a failure to down-regulate Gata2, which is normally repressed by Hes1. This study demonstrated that Gata2 levels also need to be tightly and temporally regulated, as a Notch1-induced Gata2 expression spike is essential for haematopoietic development, but sustained Gata2 expression renders haematopoietic progenitors non-functional.

Even though Notch1 signalling is required to initiate expression of important haematopoietic regulators such as Gata2, its activity also needs to be restricted to allow for the completion of the EHT, as high Notch activity promotes an arterial programme in endothelial cells, while suppressing the haematopoietic programme [54]. In fact, a reporter system that differentially labels cells with high or low Notch activity has identified two populations in the dorsal aorta, an arterial-restricted population with high Notch activity and a haematopoietic-competent population with low Notch activity [55]. That same study further revealed the involvement of two different Notch ligands, with Dll4 inducing high Notch activity and thus implementing the arterial programme, while Jag1 facilitated the haematopoietic programme by restricting Notch activity. A down-regulation of Notch signalling was also observed during the maturation of pre-HSCs into fully competent HSCs [56].

Another transcriptional regulator with a function analogous to Notch is Sox17. It is known to determine arterial fate [57], but also to specify HECs [58]. Like Notch, it promotes the endothelial programme over the haematopoietic programme and needs to be down-regulated for the EHT to proceed [54]. It was found to be co-expressed with Runx1 in HECs and has been suggested to repress Runx1 function [54,58,59]. Indeed, relative levels of Runx1 and Sox17 as determined by measuring the ratio of their mean fluorescence intensities by immunofluorescence microscopy and correlating this with morphological changes detected by scanning electron microscopy can identify cells at the different stages of EHT [59]. Flat and elongated endothelial cells that are firmly integrated into the endothelial layer had a very low Runx1:Sox17 ratio, whereas cells inside the intra-aortic clusters scored with a high Runx1:Sox17 ratio. Thus, the levels of Runx1 and Sox17 are negatively correlated, with an increasing ratio of Runx1:Sox17 associated with the adoption of a haematopoietic fate. Interestingly, this was also associated with the appearance of membrane protrusions, the significance of which needs to be determined.

Very recently, Gata2 levels during the EHT were reported to be highly dynamic as measured with the help of a short-lived fluorescent Venus reporter for Gata2 expression [60]. Not only were different levels of Gata2-Venus detected among endothelial cells and intra-aortic cluster cells, but the expression of the reporter in these cells demonstrated a highly dynamic and pulsatile nature that could be correlated with different stages along the EHT. Specifically, cells that were emerging from the endothelium to form intra-aortic clusters displayed a shorter periodicity of Gata2-Venus expression pulses than HECs, thus demonstrating a higher dynamic nature. In addition, cells rounding up at the initiation of the EHT also demonstrated higher pulse amplitudes. The significance of these Gata2 expression pulses is currently unknown, although a functional role is suggested by an alteration in pulsatile behaviour in Gata2 heterozygous embryos that have reduced haematopoietic activity.

## RNA modifications—another level of regulation

A new mode of transcriptional control that has received a lot of attention lately and also has an impact on haematopoietic cell generation in the AGM involves RNA modifications. The most common RNA modification in eukaryotes is the creation of *N*^6^-methyl-adenosine, which is laid down by RNA-specific methyltransferases, such as Mettl3 [61,62]. These modifications are recognised by specific reader proteins, which then target these RNA molecules for further processing, such as splicing, or degradation. Mettl3 was found to be expressed in endothelial cells, including HECs, and haematopoietic cells in both zebrafish and mouse embryos [63]. Furthermore, morpholino-mediated knock-down of *mettl3* in zebrafish embryos resulted in decreased *runx1* and *cmyb* expression and reduced numbers of HECs and HSPCs, which were further impaired in their haematopoietic differentiation. Interestingly, vascular development was normal, but endothelial cells maintained their endothelial identity and could not undergo the EHT. This was to a large part due to increased levels of Notch pathway components, whose mRNAs are normally targets of mettl3 and become *N*^6^-methylated at the time of EHT. Morpholino targeting of the *N*^6^-methyl-adenosine reader *ythdf2*, which targets methylated mRNAs for degradation, resulted in a similar phenotype and demonstrated that down-regulation of Notch activity for completion of EHT is, at least in part, achieved by modulation and degradation of *notch1a* mRNA via mettl3 and ythdf2. This pathway is conserved in mice [63,64].

Notch activity is also down-regulated by Gpr183 signalling-mediated ubiquitination and degradation of the Notch1 protein [65].

## Molecular details of EHT

Despite having known about the existence of EHT in the generation of HSPCs for a considerable amount of time and having identified some of the key transcriptional regulators and signalling pathways, knowledge of the molecular details, especially the morphological changes and restructuring involved, is still very much limited. Many transcriptional profiling studies on non-HECs, HECs, cells undergoing EHT, intra-aortic cluster cells and HSPCs, some even at the single-cell level, have been performed in recent years with the aim of shedding more light on this process [49,66–71] (Table 1).

**Table 1:** Phenotype of cell populations analysed by transcriptional profiling

Cell population	Phenotype	Stage	Profiling	Reference
Non-HECs HECs HSPCs	Runx1 + 23GFP^−^Cdh5^+^Ter119^−^CD45^−^CD41^−^ Runx1 + 23GFP^+^Cdh5^+^Ter119^−^CD45^−^CD41^−^ Runx1 + 23GFP^+^Cdh5^+^Ter119^−^CD45^−^CD41^+^	E8.5, E9.25, E10.5	Microarrays and scqRT-PCR	[49]
Non-HECs HECs HSCs HPCs	Ly6a-GFP^−^CD31^+^ckit^−^ Ly6a-GFP^+^CD31^+^ckit^−^ Ly6a-GFP^+^CD31^+^ckit^+^ Ly6a-GFP^−^CD31^+^ckit^+^	E10.5	Bulk RNA-Seq	[70]
GFP^+^ ECs GFP^−^ ECs GFP^+^ HCCs GFP^−^ HCCs	Ly6a-GFP^+^CD31^+^Cdh5^+^Esam^+^ckit^−^ Ly6a-GFP^−^CD31^+^Cdh5^+^Esam^+^ckit^−^ Ly6a-GFP^+^CD31^+^Cdh5^+^Esam^+^ckit^+^ Ly6a-GFP^−^CD31^+^Cdh5^+^Esam^+^ckit^+^	E10.5	Bulk RNA-Seq	[68]
Trunk HECs Trunk rest	*runx1*-GFP^+^ *runx1*-GFP^−^	26–28 hpf	Bulk RNA-Seq	[67]
ECs Pre-HSC I Pre-HSC II HSC (FL) HSC (FL)	CD31^+^Cdh5^+^CD41^−^CD43^−^CD45^−^Ter119^−^ CD31^+^CD45^−^CD41^lo^cKit^+^EPCR^hi^ CD31^+^CD45^+^CD41^lo^ Lin^−^Sca1^+^Mac1^lo^EPCR^+^ CD45^+^CD150^+^CD48^−^EPCR^+^	E11.5 E11.5 E11.5 E12 E14	10-cell and scRNA-Seq	[71]
HCCs Non-HECs HECs EHT cells Pre-HSC I Pre-HSC II HSPCs (YS)	ckit^+^ Cdh5^+^Gfi1-tomato^−^Gfi1b-GFP^−^ckit^−^ Cdh5^+^Gfi1-tomato^+^Gfi1b-GFP^−^ckit^−^ Cdh5^+^Gfi1-tomato^+^Gfi1b-GFP^−^ckit^+^ Cdh5^+^ckit^+^CD45^−^ Cdh5^+^ckit^+^CD45^+^ ckit^+^	E11.5 E10/E11 E11 E10/E11	scRNA-Seq	[66]
ECs-HSPCs Non-HECs HECs Pre-HSPC I Pre-HSPC II Pro-HSCs Pre-HSC I Pre-HSC II Non-HECs HECs Pre-HSPC I Pre-HSPC II	Cdh5^+^ Cdh5^+^CD44^−^ Cdh5^+^CD44^lo^ckit^−^ Cdh5^+^CD44^lo^ckit^+^ Cdh5^+^CD44^hi^ Cdh5^+^CD41^+^CD43^−^CD45^−^ Cdh5^+^CD41^+^CD43^+^CD45^−^ Cdh5^+^CD41^+^CD43^+^CD45^+^ Cdh5^+^CD44^−^ Cdh5^+^CD44^lo^ckit^−^ Cdh5^+^CD44^lo^ckit^+^ Cdh5^+^CD44^hi^	E10.5 E10 E10 E9.5/10/11 E9.5/10 E11	scRNA-Seq scqRT-PCR scqRT-PCR Bulk RNA-Seq	[69]

Abbreviations: EC, endothelial cell; EHT, endothelial-to-haematopoietic transition; FL, foetal liver; HEC, haemogenic endothelial cell; HCC, haematopoietic cluster cell; HPC, haematopoietic progenitor cell; hpf, hours post fertilisation; HSC, haematopoietic stem cell; HSPC, haematopoietic stem/progenitor cell; sc, single cell; YS, yolk sac.

One of the first profiling studies of the EHT took advantage of the previously identified Runx1 + 23 enhancer, which is active in definitive HSPCs and HECs and, when linked to a GFP reporter, serves as an excellent tool for the isolation of these populations [49,50,72]. Through a combination of expression analyses and functional assays, it was established that HECs gradually lose their endothelial identity between E8.5 and E10.5 as their haematopoietic potential increases, with clonogenic assays indicating that endothelial and haematopoietic capacities are mutually exclusive [49]. The initiation of the haematopoietic programme in endothelial cells destined to become HECs is co-ordinated by Runx1 and the rapid decline of Runx1 expression in endothelial cells after E10.5 points to a very transient nature of HECs.

Other studies have focused on one or two developmental time points and compared the different cell populations from non-HEC to HSPCs with the aim of identifying, and ordering along a differentiation trajectory, the intermediate cellular states with the accompanying transcriptional stages. The identification of additional cell surface markers [69,71] and the use of further fluorescent reporter mouse lines [66,68,70] have made these isolation strategies ever more sophisticated.

Using a combination of markers for endothelial cells (CD31), intra-aortic cluster cells (ckit) and HECs/HSPCs (Ly6a-GFP), non-HECs, HECs, HSCs and more mature haematopoietic cells were compared, which led to the identification of the G protein-coupled receptor Gpr56 as being up-regulated concomitant with the onset of the haematopoietic programme, with functional studies in zebrafish confirming a role for Gpr56 in HSC emergence [70]. This study also reported an up-regulation of genes in HECs associated with migration, Notch pathway activation and hypoxia. Indeed, the same group had previously described a role for the hypoxia-induced gene Hif1a in HSC development [73]. Interestingly, a cell cycle signature was not detected until the HSC state, suggesting that cell proliferation occurs after completion of haematopoietic differentiation [70].

Using the same Ly6a-GFP reporter line, but in combination with CD31, Cdh5, Esam and ckit, another group compared GFP+ and GFP− endothelial cells and intra-aortic cluster cells [68]. They described a striking up-regulation of inflammatory pathways in HECs that was not triggered by an infection. Specifically, they found that interferon signalling increased HSPC production. This novel role of sterile pro-inflammatory signals promoting HSPC emergence from HECs was also discovered by two other groups using the zebrafish system [67,74], who reported that TNFα produced by primitive neutrophils initiated an inflammatory response in HEC via Tnfr2 and the TLR4–MyD88–NFκB axis, with the Notch pathway being an important downstream target. The activation of this cascade was required for the specification of haematopoietic cells from HECs.

The advent of single-cell RNA-Seq has allowed a more detailed dissection of the differentiation pathway from endothelial cell to haematopoietic cell [66,69,71]. Additional intermediate cellular states have been uncovered that can be ordered along a differentiation trajectory using pseudotime algorithms, which is most prominently marked by a gradual down-regulation of endothelial-specific genes and an up-regulation of haematopoietic genes. Zhou et al. described EPCR as a cell surface marker up-regulated as endothelial cells differentiate into pre-HSCs [71], with another group describing a further enrichment for pre-HSCs through the combination of EPCR with Dll4 [75]. This differential expression of EPCR between endothelial cells and pre-HSCs allowed Zhou et al. to isolate and sequence single endothelial cells, type I and II pre-HSCs and foetal liver HSCs [71]. They noticed the biggest transcriptional change taking place during the transition from endothelial cell to pre-HSC, with a general increase in transcriptional activity in pre-HSCs, which included genes involved in mitochondrial aerobic respiration, in the mTOR pathway and also inflammation-related genes. In addition, a high degree of heterogeneity in cell cycle status in the pre-HSC population was observed. Indeed, it was recently described that intra-aortic cluster cells at an earlier maturation stage, located at the base of the cluster, are more quiescent than more mature cells found at the tip of the cluster which are more actively cycling [76]. This suggests an expansion of pre-HSCs prior to their colonisation of the foetal liver and maturation into HSCs. The same authors have subsequently mined their data sets specifically for long non-coding RNAs and detected an up-regulation of H19 at the pre-HSC stage. An endothelial-specific knockout of the differentially methylated region that controls H19 expression led to severely impaired EHT and formation of HSPCs. Mechanistically, H19 was shown to regulate promoter methylation of key EHT transcription factors, such as Runx1, with increased methylation in its absence causing a down-regulation of Runx1 [77].

Single-cell RNA-Seq profiling by Baron et al. also highlighted that the greatest number of transcriptional changes takes place during the EHT, while there were only few genes differentially expressed during the transition from a non-HEC to an HEC [66]. The authors also carried out a careful analysis of the composition of the intra-aortic clusters and found these to contain type I and type II pre-HSCs as well as more committed HSPCs. This study has sequenced the most comprehensive repertoire of cell populations from endothelial cells to HSPCs to date. With gene sets specifying each step having been identified, this should serve as an important resource.

In the most recent single-cell RNA-Seq study, CD44 was identified as a marker that becomes detectable specifically in the transitioning population in which endothelial and haematopoietic programmes are co-expressed. Its levels further increase with the onset of ckit expression and mark cells that undergo morphological changes during the EHT. In fact, together with ckit and Cdh5, CD44 allowed an accurate isolation of cell stages from non-HEC to mature HSPCs. Furthermore, CD44 appears to have a functional role, as a disruption of the interaction with its ligand hyaluronan interfered with the EHT. It was further noted that compared with non-HEC, HECs were less proliferative and displayed a lower metabolic activity, but exhibited an increase in the expression of genes linked to lipid biosynthesis, Notch pathway activation and autophagy. An exit from the cell cycle may be necessary for a cell to undergo the EHT.

## EHT vs. epithelial-to-mesenchymal transition

On the surface, EHT seems to share similarities with another major cell fate change — the epithelial-to-mesenchymal transition (EMT), and it was this obvious analogy that led to the adoption of the term EHT. Like EHT, EMT plays an essential role during development in tissue morphogenesis, but it is also a process involved in metastasis, when tumour cells gain the ability to leave their tissue-of-origin and seed other sites in the body. It is interesting in this context that it was recently reported that homophilic interactions of CD44 on breast cancer cells enhance the formation of clusters of these cells, which leads to increased metastatic potential [78]. It should be investigated whether CD44 may also be important for intra-aortic cluster formation during EHT and what the functional significance of this may be.

Another pathway that is shared by the EMT and EHT is Tgfb signalling [37,79–83]. Activation of the Tgfb pathway was shown to promote the EMT, while its role in the EHT has been seemingly controversial. While some reports suggested that activation of Tgfb signalling promotes the EHT [81,82], others suggested that Tgfb activity needs to be down-regulated for the EHT to proceed [37,80,83]. While some of the controversies may be explained by the suggestion that the compound SB431542, commonly employed as an inhibitor of the Tgfb pathway [37,83], may have a more complex mode of action and can in fact increase phosphorylation and activation of Smad2/3 [82], which are downstream effectors of Tgfb signalling, other reports suggest that Tgfb signalling, like Notch activation, is essential for the specification of HECs, but may have to be down-regulated for the EHT to proceed [80]. In this context, the role of HDAC1 may also need further clarification. It is clear that it is required for HSPC specification from endothelial cells; however, in one study, it was reported to act by modulating the Tgfb pathway and Smad2/3 [82], while another group found it to activate Smad1/5, which led to ERK inhibition and the promotion of the haematopoietic programme [84]. A similar controversy surrounds the related Bmp signalling pathway, which may also have to be down-regulated during the final stages of HSPC formation [85].

## Remaining questions

Our understanding of the details of the EHT has increased substantially in recent years (Figure 2), aided by the development of more sophisticated profiling and analysis tools. Despite this increase in knowledge, routine and efficient generation of HSCs from PSCs has still not been achieved although recent progress is promising. What are we still missing?

The more we learn about the process of HSPC specification, the more complex it turns out to be with many unanswered questions remaining. Haematopoietic progenitor generation from PSCs is much more successful, so how does it differ from producing HSCs? The embryo generates both stem and progenitor cells from HEC, but the former is a much rarer event, and we do not currently know how these two processes differ and why an HEC in one case may give rise to a stem cell, while in other cases, a more mature progenitor is produced. There is also evidence that there are different types of HECs [86], and that the process of the EHT may not be the same in every haematopoietic tissue [16]. Related to this is the question of how HECs are specified in the first place. While grafting experiments in avian embryos suggested a spatial segregation of haemogenic and non-haemogenic-fated endothelial precursors early on in development [87], it was more recently suggested that all nascent endothelial cells are competent to become HECs [49].

Even though there is very good evidence from endothelial fate mapping and endothelial-specific knockout studies (described above) that HSCs are generated from HECs, it could nevertheless be speculated that HSCs are in fact generated via a different mechanism—one that has so far not been recreated in the PSC system. Some of the markers that are employed for endothelial-specific targeting, such as Cdh5, continue to be expressed on HSCs, and temporal control of expression *in vivo* can be challenging [88]. Some staining results suggest that HSC precursors may also be located in the sub-aortic mesenchyme [30], and a knockout of Runx1 resulted in the accumulation of Runx1-LacZ+ cells in the sub-endothelial layer [89]. Whether these HSC precursors, upon maturation to HSCs, directly traverse the endothelial layer, temporarily integrate into the endothelial layer or adopt an endothelial fate before undergoing EHT, requires further investigation.

We also know relatively little about the surrounding supportive cells that make up the microenvironment and how they promote the EHT. It is clear that there is something special about the AGM microenvironment that allows HSC specification to occur in this part of the vasculature so efficiently. We are slowly starting to learn more about these AGM-derived supportive signals [90,91].

It is also clear that a simple activation of key transcription factors is not sufficient, but that the timing, co-expression, levels and dynamics need to be precisely controlled. These are all challenges that may have to be overcome before we can rely on a patient-specific supply of *in vitro*-generated HSCs for the clinic.

## Perspectives

- The EHT is the process through which haematopoietic stem and progenitor cells are first generated during development. A deciphering of the details would allow blood generation from pluripotent stem cells *in vitro* for cell replacement therapies.
- Several of the key transcription factors and signalling pathways driving EHT have been identified. Intermediate cellular and transcriptional states are being elucidated through novel *ex vivo* culture systems and expression profiling.
- More insight into the timing, relative levels and dynamics of key transcription factor expression as well as the contribution of the microenvironment need to be gained before robust generation of haematopoietic stem cells *in vitro* can be achieved.

## Abbreviations

AGM: aorta-gonads-mesonephros
EC: endothelial cell
EHT: endothelial-to-haematopoietic transition
EMPs: erythroid-myeloid progenitors
EMT: epithelial-to-mesenchymal transition
HCC: haematopoietic cluster cell
HEC: haemogenic endothelial cell
HPC: haematopoietic progenitor cell
HSC: haematopoietic stem cell
HSPC: haematopoietic stem/progenitor cell
PSCs: pluripotent stem cells
iPSCs: induced PSCs
